# Photocatalytic Activities of g-C_3_N_4_ (CN) Treated with Nitric Acid Vapor for the Degradation of Pollutants in Wastewater

**DOI:** 10.3390/ma16062177

**Published:** 2023-03-08

**Authors:** Ruishuo Li, Bingquan Wang, Rui Wang

**Affiliations:** 1 School of Environmental Science and Engineering, Shandong University, Qingdao 266237, China; 2 School of Chemistry and Molecular Engineering, Qingdao University of Science and Technology, Qingdao 266042, China

**Keywords:** g-C_3_N_4_, photocatalytic activity, rhodamine B, tetracycline hydrochloride, degradation, wastewater

## Abstract

In this article, we reported a novel setup treatment using nitric acid vapor to treat g-C_3_N_4_ (CN). By treatment with nitric acid vapour, the basic structure of the CN has not been destroyed. These adoptive treatments enhanced the photocatalytic performance of CN and were reflected in the elimination of rhodamine B (RhB) as well as tetracycline hydrochloride (TC). In comparison to CN, CN-6 demonstrated the highest photocatalytic yield for the breakdown of RhB (99%, in 20 min). Moreover, the excellent reuse of CN-6 for breaking down RhB was also demonstrated. This clearly demonstrated that treatment with nitric acid vapor promoted a blue shift, positively extended its valence band position, and increased the oxidizability of the holes. This also caused CN to disperse better into the aqueous phase, introducing more oxygen-containing functional groups. Thus, treatment with nitric acid vapor has the potential to be applied to delaminate the CN in order to enhance photocatalytic activity.

## 1. Introduction

Due to industrialization and population growth, many countries have critical environmental issues, such as energy crises and pollution of the environment [1]. Nowadays, the development of state-of-the-art technologies to address these issues at a global scale is of great importance [2,3]. Among various proposed technologies, photocatalytic technology has tremendously gained global attention to alleviate environmental contamination because of its ability to make direct use of inexhaustible solar power [4,5,6]. Although one of the earliest investigated photocatalysts is titanium dioxide (TiO_2_), it exhibits significant limitations due to the large band gap and responds only to ultraviolet light [7,8]. As a visible light active photocatalyst, metal-free graphitic carbon nitride (g-C_3_N_4_) has recently attracted a lot of attention. It has distinctive properties for photocatalysis, such as a tunable band gap (2.7 eV), single optical properties to harvest sunlight, high stability to resist photo-corrosion, suitable electronic properties, environmental friendliness, and low cost [9,10,11,12,13]. Though the g-C_3_N_4_ exhibited immense potential in the photocatalysis field, it still suffers from poor active sites, a low redox potential of photogenerated charge carriers, a low quantum yield, and a poor dispersion of the aqueous phase [14,15].

Several modifications have been developed to ameliorate the photocatalytic performance of the bulk g-C_3_N_4_, such as element doping [16,17,18], heterojunction building [19,20], and morphology controlling [21,22]. Motivated by the pioneering work on graphene [23], many researchers have made efforts to delaminate the bulk g-C_3_N_4_ into 2D thinner counterparts and explore their novel properties. The ultrathin 2D g-C_3_N_4_ nanosheet has distinct physicochemical properties that set it apart from bulk g-C_3_N_4_, such as a broader bandgap, a higher redox ability of charge carriers, an open-up surface, and an extended life for charge carriers. These properties give g-C_3_N_4_ nanosheets improved photocatalytic performance in real-world applications [24,25,26].

Niu and his coworkers first developed g-C_3_N_4_ nanosheets by long-time thermal oxidation exfoliation that increased bandgap and ameliorated the capacity of electron transport. The super photocatalytic activities of g-C_3_N_4_ nanosheets as photocatalysts were established via H_2_ evolution [27]. Yan et al. successfully exfoliated g-C_3_N_4_ in hot water to obtain ultrathin g-C_3_N_4_. The as-prepared g-C_3_N_4_ ultrathin has a thickness of 1.2 nm after 12 h of treatments, with a wide lateral dimension and improved photoactivity for the degradation of organic dye when exposed to visible light [28]. Miao et al. synthesized 2D g-C_3_N_4_ nanosheets with acid treatment that demonstrated an excellent photocurrent response close to 0.5 mA/cm^2^ and lower charge transfer resistance [29]. Although the majority of the currently used techniques (such as thermal exfoliation, liquid etching, and chemical exfoliation using potent oxidizing acids) can exfoliate bulk g-C_3_N_4_ into a single layer, they are also ineffective because the tri-s-triazine units are destroyed, the processing time is prolonged, and the yield of nanosheets is subpar [30]. Therefore, it is advisable to develop an advanced strategy with high efficiency and low energy consumption for delaminating bulk g-C_3_N_4_ to ultrathin g-C_3_N_4_ nanosheets.

Herein, we present a simple process to prepare g-C_3_N_4_ nanosheets through nitric acid vapor treatment using a new practical carbon material processor invented by our research group. Compared with aqueous exfoliation, the process has the merits of a controllable treatment time, avoiding over-oxidation, easy collection of products, and high yield. The obtained nanosheets were studied according to the crystal structure, surface group, composition, and physicochemical properties. The g-C_3_N_4_ nanosheets were found to exhibit a stronger oxidizing capacity and disperse well into an aqueous solution. As anticipated, when exposed to visible light, g-C_3_N_4_ nanosheets demonstrated outstanding photocatalytic activity and stability for the destruction of RhB and TC. Moreover, a proper mechanism and the active species for the degradation of RhB were also studied through scavenger experiments.

## 2. Materials and Methods

### 2.1. Materials

Urea was bought from Bodi (Tianjin, China). Tetracycline hydrochloride (TC) was obtained from Shanghai Ruiyong Biotechnology Co., Ltd (Shanghai, China). Triethanolamine (TEOA), concentrated nitric acid and absolute ethanol were purchased from Fuyu (Tianjin, China). Rhodamine B (RhB) was purchased from Kemiou (Tianjin, China). p-Benzoquinone (p-BQ) was purchased from Shanghai Macklin Biochemical Co. Ltd (Shanghai, China). All reagents were of analytical grade and used with no further purification.

### 2.2. Synthesis of the Photocatalysts

The bulk g-C_3_N_4_ was prepared using the method reported previously [31]. In a nutshell, 20.0 g of urea was placed in a 100 mL crucible with a cover slip and heated in a muffle furnace to 550 °C for 3 h at a rate of 17.8 °C/min. After being allowed to cool to ambient temperature, the resulting yellow substance was ground into a powder in an agate mortar. The synthesized sample was denoted as CN.

**Figure 1:**
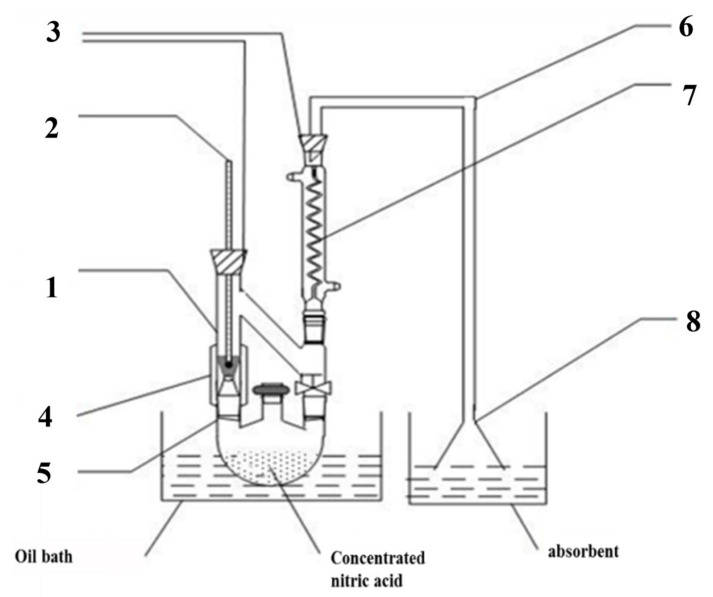
Self-made HNO_3_ vapor treatment device ((1): steam treatment device, (2): thermometer, (3): rubber plug, (4): heating belt, (5): three-mouth flask, (6): catheter, (7): snake condensation tube, (8): funnel).

The alteration to the bulk g-C_3_N_4_ was treated with nitric acid vapor. Some concentrated HNO_3_ was poured into a flask with three necks, and g-C_3_N_4_ was added to the processing apparatus as shown in Figure 1. The self-assembling device was then placed in a 170 °C oil bath for a period of time. According to the treatment time, the g-C_3_N_4_ was named CN-X (X = 2, 4, 6, 8 h).

### 2.3. Characterization

X-ray diffraction (XRD) patterns were obtained by a Bruker D8 ADVANCE instrument using Cu-Kα radiation (λ = 0.15405 nm, 40 KV × 60 mA) from 10° to 80° (2θ) with a scanning rate of 0.02°/s. X-ray photoelectron spectroscopy (XPS) was obtained by a Thermo ESCALAB 250XI with a monochromated Al Ka X-ray source. A Bruker ALPHA-T Fourier transform infrared spectrometer was used to record the Fourier transform infrared spectra (FT-IR) of the samples, with a range from 400 to 4000 cm^−1^ using the KBr pellet at room temperature. Brunauer–Emmett–Teller (BET) measurement was used to determine the specific surface area of samples (Quantachrome Instruments Quadrasorb SI, United States Quantachrome Co., Ltd, Boulder, CO, USA). Using a 100 kV accelerating voltage, a field emission scanning electron microscope (SEM) (JSM-6700) was used to characterize the morphologies of the samples. A UV-vis spectrophotometer was used to generate diffuse reflectance spectra (DRS) (SHIMADZU, UV-2550). Photoluminescence (PL) spectroscopy was performed at room temperature using a Hitachi F-4500 fluorescence spectrophotometer.

### 2.4. Photocatalytic Performance Tests

#### 2.4.1. Photocatalytic Experiments

Under visible light irradiation, TC and RhB were used as target pollutants to evaluate the photocatalytic activities of the as-prepared samples. For photocatalytic experiments, a photocatalytic reactor with a 200 mL beaker as a reaction vessel and a 300W-Xenon lamp with a 420 nm cutoff filter as a visible light source was used, with constant concurrent magnetic stirring. Typically, 25 mg photocatalysts were introduced into 100 mL RhB solution (10mg/L) and 50 mg photocatalysts were introduced into 100 mL TC solution (20 mg/ L). The mixture was then sonicated for 5 min and magnetically stirred in the dark for 30 min to achieve an equilibrium between adsorption and desorption. After a specific time interval of irradiation, 4 mL of the suspension was removed and filtered through a 0.22 µm filter membrane to remove the photocatalyst. A UV-vis spectrophotometer was used to measure the concentrations of TC and RhB. (SP-756PC, Shanghai Spectrum Instruments Co., Ltd, Shanghai, China).

#### 2.4.2. Determination of Active Species during Photocatalytic Degradation

Radical trapping experiments were performed to ensure the presence of active species in the RhB photo-degradation process. In this study, p-Benzoquinone (p-BQ), triethanolamine (TEOA), and isopropanol (IPA) were used as scavengers of holes (h^+^), hydroxyl radicals (•OH), and superoxide radicals (•O_2_^−^), respectively. Prior to reaching the adsorption equilibrium, the capture agents were added to the RhB solution (10 mg/L) and agitated for 30 min without light. The photocatalytic reaction experiment was then performed.

## 3. Results and Discussion

### 3.1. Characterization of the As-Prepared Photocatalysts

#### 3.1.1. Structure and Morphology Characterizations

**Figure 2:**
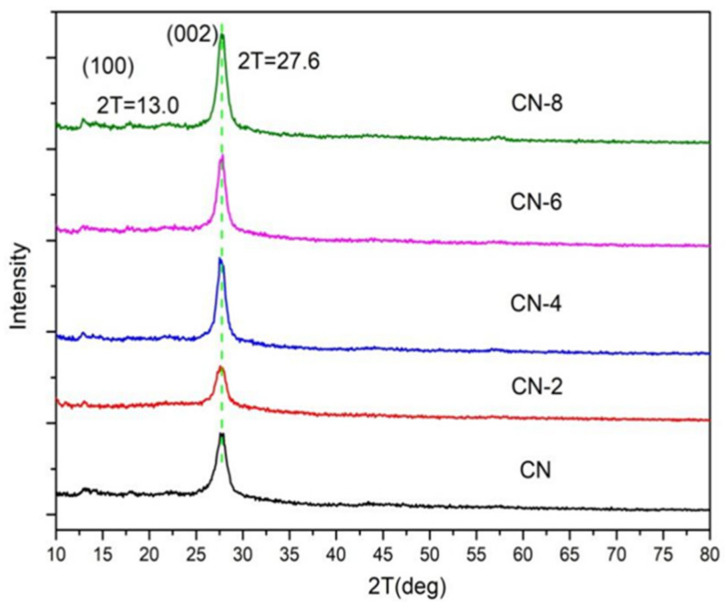
XRD patterns of the samples.

**Figure 3:**
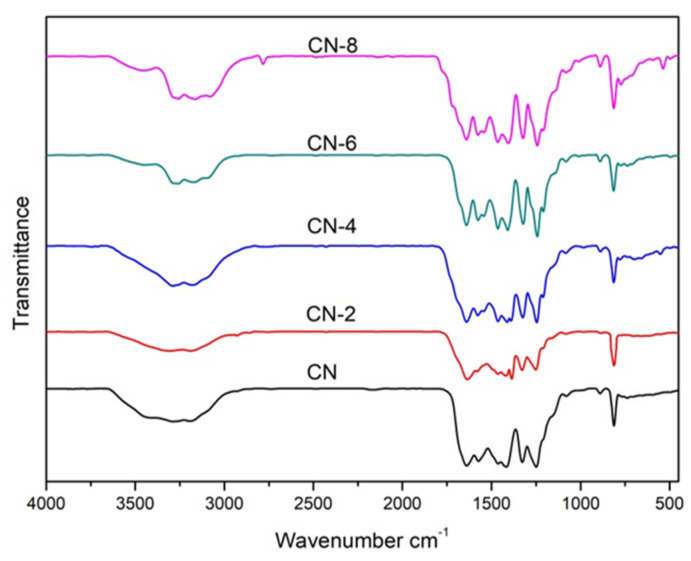
FT-IR spectra of samples.

Figure 2 displays the XRD patterns of the samples to examine the impact of HNO_3_ vapor on the crystalline phase of g-C_3_N_4_. The two distinct peaks at 12.97° and 27.59° for all samples were the in-plane (100) diffraction reflecting the interlayer stacking of the g-C_3_N_4_ aromatic units and the interlayer (002) diffraction reflecting the structural packing pattern of the aromatic segments in the plane, respectively [32]. Nitric acid steam treatment improved the interlayer stacking interaction of the aromatic conjugated system by gradually increasing the (002) peak intensity of g-C_3_N_4_ in comparison to untreated g-C_3_N_4_. This shows that nitric acid steam treatment can break the hydrogen bond between g-C_3_N_4_ layers and cause the g-C_3_N_4_ layers to peel off into nanostructures [33]. Henceforth, this confirms that the crystalline phase of g-C_3_N_4_ is unaffected following treatment with nitric acid vapor. Fourier transform infrared (FT-IR) spectra were used to study the structural information of pure and processed g-C_3_N_4_. Figure 3 demonstrates that the C-N heterocycles’ stretching vibrations at 1200–1650 cm^−1^ and the triazine units’ distinctive breathing vibration at 810 cm^−1^ were present in all the samples, indicating that the tri-s-triazine units’ chemical core structure is resistant to HNO_3_ vapor erosion. However, as the bulk of g-C_3_N_4_ was exfoliated, both the N-H and O-H stretching vibrations steadily increased, indicating that the increased density of the −OH group in g-C_3_N_4_ was caused by the acid hydrolysis action of the bridging N groups [34].

**Figure 4:**
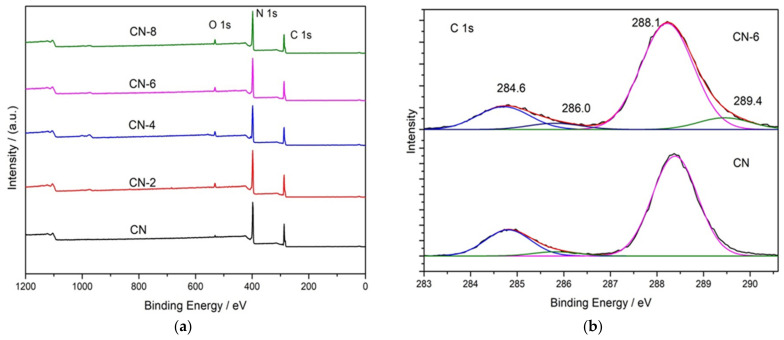

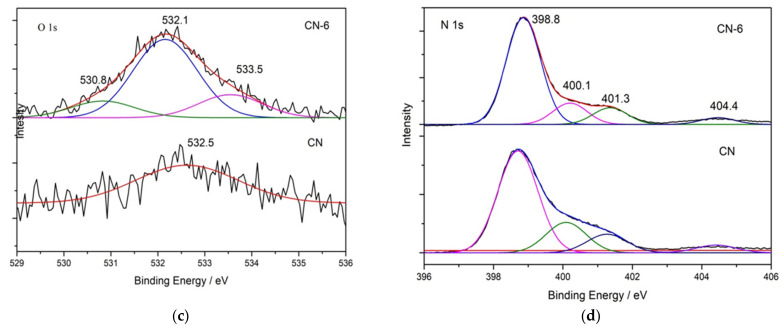
XPS survey of all samples (**a**) and high-resolution spectra in C 1 s (**b**), O 1 s (**c**), and N 1 s (**d**); binding energy regions of CN and CN-6.

X-ray photoelectron spectroscopy (XPS) was used to determine the elemental surface composition and chemical state of g-C_3_N_4_ (Figure 4). The XPS spectrum in Figure 4a indicates that all the samples are mainly composed of C, N, and O. Figure 4b shows the high-resolution XPS spectrum of C 1s for the bulk g-C_3_N_4_ and treated g-C_3_N_4_, in which the four peaks at 284.6 eV, 286.0 eV, 288.1 eV and 289.4 eV can be attributed to C = C, C-NH_2_, N-C = N, and C-O [35]. When CN-6 was compared to bulk g-C_3_N_4_, the intensity of the peak at 286.0 eV and 289.4 eV was increased. This could be attributed to C-O groups formed during the acid hydrolysis of the tri-s-triazine unit, which bridged C-N-C [36]. The intensity of the peak at 286.0 eV and 289.4 eV increased when CN-6 was compared to bulk g-C_3_N_4_. C-O groups formed during the acid hydrolysis of the tri-s-triazine unit, which bridged C-N-C, could be responsible for this. Figure 4c displays the XPS spectra for O 1s of the bulk g-C_3_N_4_ and CN-6 catalysts. The asymmetrical O 1s spectra of the two samples could be deconvoluted into these components at 533.5 eV, 532.1 eV, and 530.8 eV, attributable to the chemisorbed water, surface-adsorbed oxygen species, and surface lattice oxygen, respectively. Interestingly, the N 1s high-resolution spectrum (Figure 4d) revealed three typical peaks at 398.8 eV, 400.1 eV, and 404.4 eV corresponding to the C-N = C, −NH_2,_ and graphitic species, respectively [37]. The XPS results show the stability of g-C_3_N_4_ preserved even after nitric acid vapor treatment.

**Figure 5:**
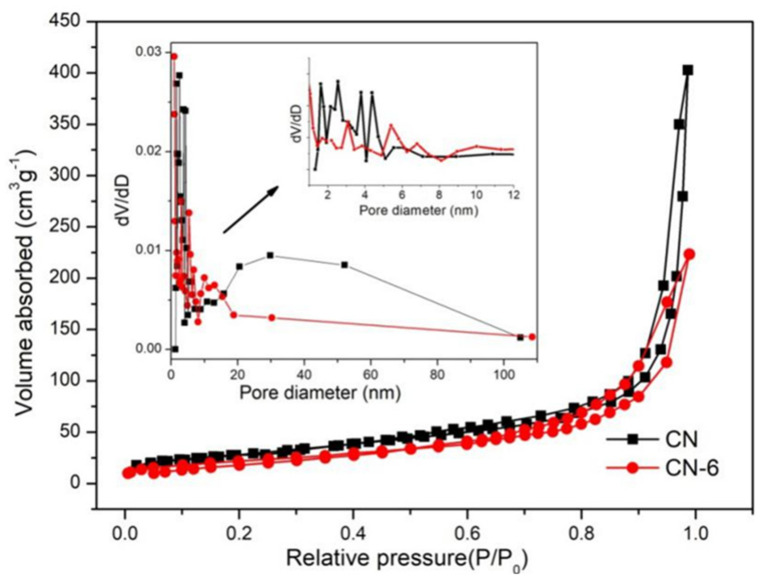
Adsorption–desorption isotherm of CN and CN-6.

**Figure 6:**
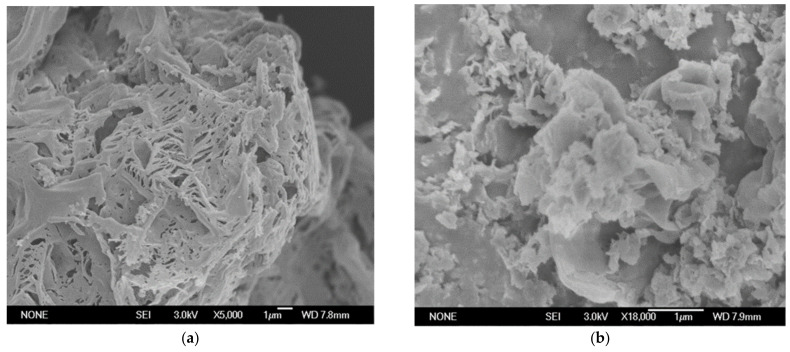
SEM images of (**a**) CN; (**b**) CN-6.

The surface characteristics of samples from the N_2_ adsorption–desorption isotherms (Figure 5) were similar. A type-IV isotherm with a hysteresis loop was obtained in both the pure and treated g-C_3_N_4_, indicating the presence of mesoporous structures. CN-6’s BET surface areas decreased by 25.7 m^2^/g when compared to bulk g-C_3_N_4_ (105.6 m^2^/g), and its pore volume decreased by 0.278 ccg^−1^. This is attributed to the bulk g-C_3_N_4_ being exfoliated to nanosheets, then the partially completed nanosheets begin to aggregate and stack [38]. Meanwhile, the original macropore was destroyed [33]. The morphology of pure and treated g-C_3_N_4_ can be illustrated via SEM images shown in Figure 6. From Figure 6a, we can see that g-C_3_N_4_ samples with rough structures were composed of flake-like, aggregated, and different micron-sizes stacking layers, which are consistent with the typical characteristics of g-C_3_N_4_ prepared through the method of polymerization [39,40]. In Figure 6b, bulk g-C_3_N_4_ was exfoliated into nanosheets and morphologically changed to cubic and spherical shapes after nitric acid vapor treatment.

#### 3.1.2. Optical Properties of the As-Prepared Photocatalysts

**Figure 7:**
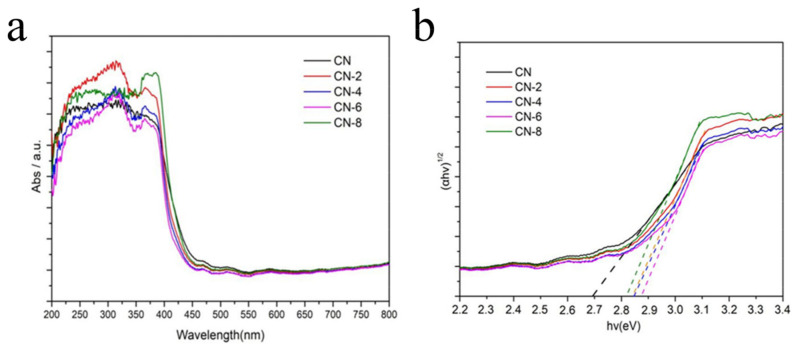
UV–vis/DRS of CN and CN-6 (**a**) and the corresponding band gap plots (**b**).

Diffuse reflectance UV-vis spectroscopy (DRS) was used to study the optical properties of the as-prepared samples. Figure 7a shows that a strong absorption band of bulk g-C_3_N_4_ was extended up to 450 nm, indicating an apparent absorption from ultraviolet to visible light. When CN-X was delaminated into nanosheets, its absorption edge had a slightly blue shift when compared to CN. CN-X, on the other hand, showed significant absorption in visible light, indicating that these samples can absorb visible light and be used for visible light photocatalysis. This absorption could be attributed to the presence of the oxygen element in the CN-X framework following acid-exfoliated CN [41]. The bandgaps (Eg) of CN and CN-X were also estimated using a plot of (αhv)^1/2^ vs. hv (*n* = 4 for indirect transition). Figure 7b shows that the Eg value increased from 2.69 eV for CN to 2.87eV for CN-6. This could be attributed to the ultrathin thickness’s strong quantum confinement effect.

**Figure 8:**
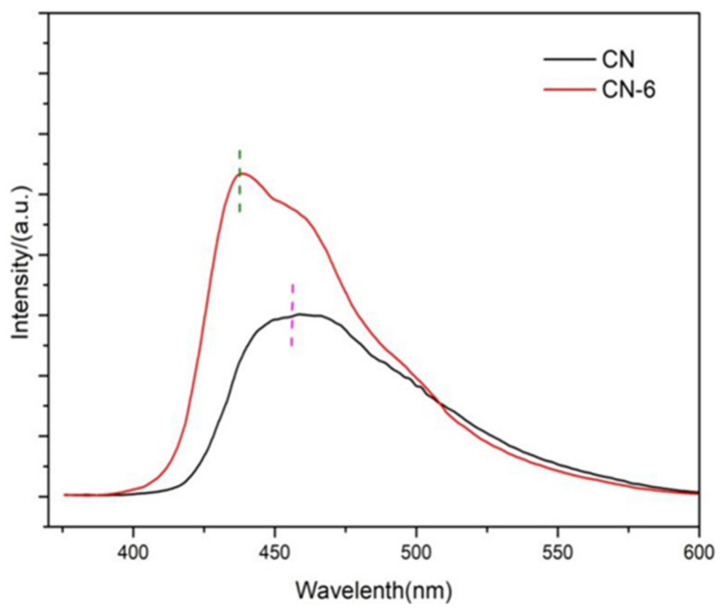
PL spectra of CN and CN-6.

It is common practice to use photoluminescence (PL) spectra as an indirect measure of charge carrier separation effectiveness. Figure 8 shows the PL spectra of g-C_3_N_4_ and CN-6. In comparison to the bulk concentration of g-C_3_N_4_, CN-6 exhibited a somewhat blue shift of the intrinsic PL peak, which corresponds to the UV-vis spectrum. Furthermore, the lower PL intensity of bulk g-C_3_N_4_ compared to CN-6, which may be due to surface defects of materials and stacking of nanosheets. It can be seen that a higher recombination rate is not the reason to improve the photocatalytic performance of the device in this paper [38].

### 3.2. Photocatalytic Activity and Stability

**Figure 9:**
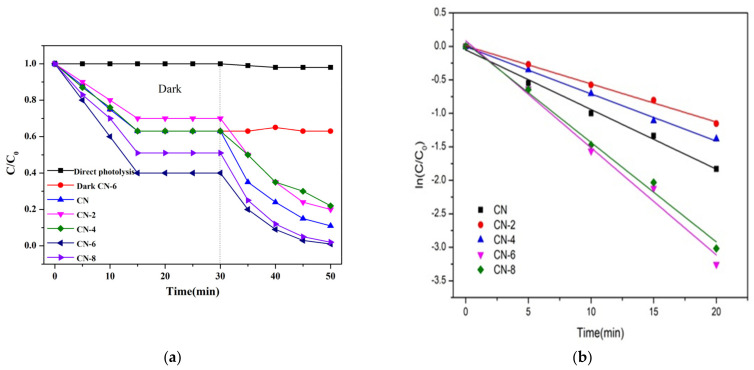

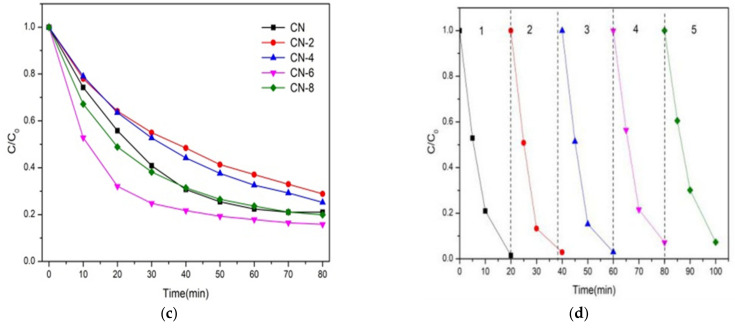
(**a**) Adsorption property and photocatalytic activity of samples towards the degradation of RhB under visible light irradiation; (**b**) the kinetic fit for RhB degradation; (**c**) photocatalytic activity of samples for TC breakdown under visible light and (**d**) during the photocatalytic breakdown of RhB over CN-6 when exposed to visible light, the photocatalytic activity cycles.

The photocatalytic performance of the as-prepared samples was assessed by the degradation of RhB and TC as a representative noxious pollutant under visible light irradiation conditions. The mixture of photocatalysts and impurities was magnetized and swirled in the dark for 30 min to create an adsorption–desorption equilibrium before being exposed to radiation. As shown in Figure 9a, the adsorption equilibrium was reached after 20 min of dark adsorption under these conditions, and we can obviously find that the adsorption capacity of CN-6 is stronger than that of CN. The large adsorption capability of the catalyst is advantageous to increase the photocatalytic performance because photocatalytic reactions take place on the catalyst surface. For comparison, a blank experiment without the photocatalyst was measured. The results in Figure 9a display that RhB can hardly decompose within 20 min of visible light irradiation, which can exclude the self-photolysis of RhB. The degradation performance values of CN, CN-2, CN-4, CN-6, and CN-8 were 89%, 80%, 78%, 99%, and 98%, respectively.

To gain insight into the photocatalytic activity of CN-X, the reaction kinetics of the degradation process were studied, and the result is shown in Figure 9b. The reaction kinetics were found to be appropriate for the pseudo-first-order model (Equation (1)), in which the slope of the fitted line can determine reaction rate constants (k).

−ln (C/C_0_) = kt (1)

where C and C_0_ are the concentrations of RhB at irradiation times t and 0, and k is the apparent reaction rate constant. As a result, the pseudo-first-order-reaction kinetics (k) of RhB degradation relative to CN-6 was 0.16, which was twice as great as that of CN (0.08). The above result may be explained by the fact that the addition of functional groups containing oxygen causes the wells to disperse in the aqueous phase, which facilitates the catalyst’s interaction with the contaminants. In addition, the absorption edge of CN-6 exhibits a somewhat blue shift after nitric acid vapor treatment, resulting in the valence band position of CN-6 becoming positively shifted and the hole potential has stronger oxidizability. In addition, to test its universality, we also employed CN-X for the photodegradation of tetracycline hydrochloride. As shown in Figure 9c, the degradation of TC by CN-X system shows the same trend as that of RhB. According to the above experiments, the catalytic performance of g-C_3_N_4_ by nitric acid vapor treatment has been confirmed. The enhancement of photocatalytic activity of g-C_3_N_4_ mainly occurs because of nanosheet delaminated and the introduction of oxygen-containing groups caused by nitric acid vapor treatment. Reusability and stability are vital for photocatalysts in the practical applications. In order to investigate the reusability of CN-6, five successive cycles were performed for photodegrading RhB under the same conditions. As shown in Figure 9d, the degradation stays almost unchanged in the second recycle, and the degradation efficiency of RhB was still as high as 93% after five cycles, demonstrating that CN-6 possessed superior reusability.

### 3.3. Discussion of Underlying Photocatalyst Mechanisms

**Figure 10:**
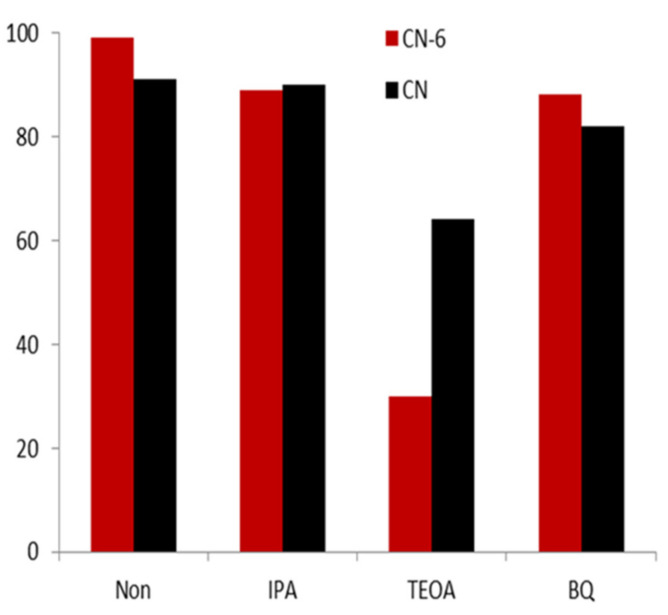
The trapping experiment of the active species during the photocatalytic degradation of RhB relative to CN-6 under visible light irradiation.

Photoactive substances such as electrons, holes, superoxide radicals, and hydroxyl radicals are expected to photo-catalyze the degradation of RhB [42]. After using various quenchers to quench a variety of active species, we then aimed to determine the influence on the photocatalytic performance of CN-6, with the purpose of identifying the main active species in the photocatalytic process. Figure 10 shows that the degradation efficiency of RhB did not have an obvious decrease compared to the result without any scavenger after the addition of 10 mmol/L of isopropanol (IPA) as the OH quencher. This demonstrated that the predominant active species in the photocatalytic reaction were not the OH radicals. The fact that the degradation efficiency was unaffected by the addition of 10 mmol/L p-Benzoquinone (p-BQ) as an ·O_2_^−^ scavenger further demonstrated that O_2_^−^ was not the main active species. This is due to the conduction band of CN-6 becoming more positive, resulting in the weaker reduction of electrons, which further affects the formation of O_2_^−^. In contrast, with the 10 mmol/L of triethanolamine (TEOA) in the form of h^+^ scavenger, the degradation efficiency of RhB decreased by 69%. This demonstrated that h^+^ was the major active species of the photocatalytic process.

**Figure 11:**
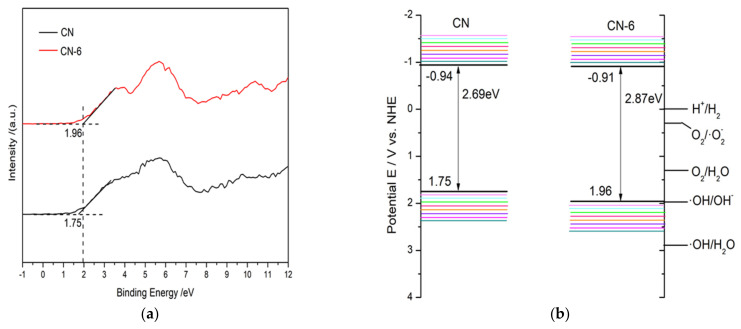
(**a**) The valence band X-ray photoelectron spectroscopy of CN and CN-6, and (**b**) band structures of CN and CN-6.

Based on the valence band minimum (VBM) roughly evaluated from the valence band X-ray photoelectron spectroscopy (Figure 11a) and Eg result from UV–vis absorption spectra, the conduction band minimum (CBM) could be roughly calculated according to the following equation: E_g_ = VBM-CBM

**Figure 12:**
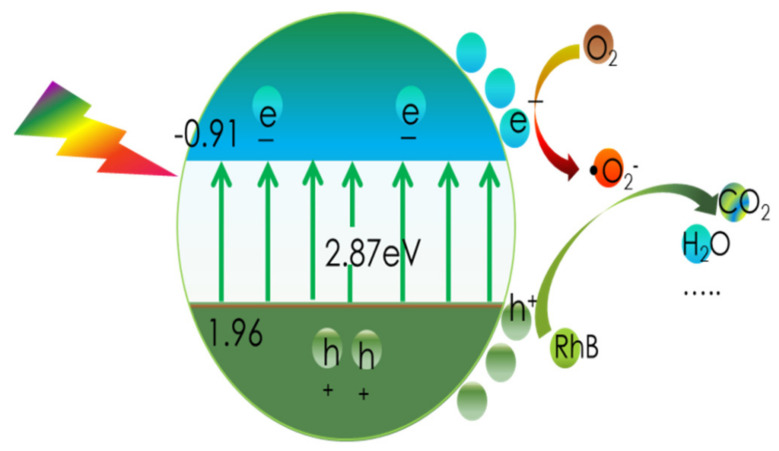
The process of RhB degradation over CN-6.

As a result, Figure 11b provides a schematic depiction of the band structures of the bulk forms of g-C_3_N_4_ and CN-6. The Eq. was expanded to 2.87 eV compared to the 2.69 eV of the bulk g-C_3_N_4_, which is attributed to the strong quantum confinement effect of the thickness of ultrathin. Correspondingly, the VBM of CN-6 was positively shifted to 1.96 eV from the 1.75 eV of bulk g-C_3_N_4_, indicating the oxidizability of the hole became stronger. Moreover, introducing oxygen-containing functional groups is said to promote visible light harvesting [28]. The process of RhB degradation over CN-6 is shown in Figure 12. In the presence of visible light irradiation, electrons on the VBM of CN-6 can be readily excited and transferred to the CBM of CN-6, thus leaving holes on the VBM. Photoinduced electrons will reduce O_2_ adsorbed to the surface to generate O_2_^−^, which would be expected to oxidize RhB in an aqueous solution. The photoinduced holes will transfer to its surface and participate in the oxidation reaction [43]. More significantly, oxygen-containing functional groups such as C-OH and C = O can emerge and develop into electrophilic groups to prevent charge carriers from recombining.

The high use of solar power, high surface area, and low recombination rate of photogenerated electron–hole pairs are widely recognized to be beneficial to the photo-activity of a photocatalyst. However, the BET result shows that CN-6’s surface area somewhat decreased, and the DRS result shows that CN-6’s absorption edge exhibited a minor blue shift after being exposed to nitric acid vapor. The high photocatalytic activity of CN-6 could thus be caused by the effective dispersal into the aqueous phase and a stronger oxidation of the hole potential. The results clearly demonstrate that nitric acid vapor treatment promoted a blue shift, positively extended its valence band position, and enhanced the oxidizability of holes.

## 4. Conclusions

In brief, CN-X was synthesized successfully by treatment with urea-pyrolysis and nitric acid vapors. CN-6 possessed excellent photocatalytic performance and stability, which is ascribed to the blue shift and the abundance of oxygen containing functional groups on surfaces after nitric acid vapor treatment. On the one hand, the introduction of oxygen-containing functional groups in the process of lamellar exfoliation can make g-C_3_N_4_ disperse better in the aqueous phase, which facilitates the contact of the photocatalyst with pollutants. On the other hand, the absorption edge of CN-6 exhibits a somewhat blue shift, and the valence band position of the CN-6 is positively shifted after nitric acid vapor treatment, which leads to the stronger oxidation of hole potential. Furthermore, the results of the trap experiment demonstrated that photodegradation is dominated by h^+^. The results of this work provide a novel method for the self-modification of bulk g-C_3_N_4_, which can enhance the photoactivity of g-C_3_N_4_ for sustainable environmental restoration.

## Data Availability

Not applicable.

## References

[1] Singh K., Nowotny J., Thangadurai V. (2013). Amphoteric oxide semiconductors for energy conversion devices: A tutorial review. Chem. Soc. Rev..

[2] Tong H., Ouyang S.X., Bi Y.P., Umezawa N., Oshikiri M., Ye J.H. (2012). Nano-photocatalytic materials: Possibilities and challenges. Adv. Mater..

[3] Kudo A., Miseki Y. (2009). Heterogeneous photocatalyst materials for water splitting. Chem. Soc. Rev..

[4] Zhang J., Chen Y., Wang X. (2015). Two-dimensional covalent carbon nitride nanosheets: Synthesis, functionalization, and applications. Energy Environ. Sci..

[5] Reddy P.A.K., Reddy P.V.L., Kwon E., Kim K.-H., Akter T., Kalagara S. (2016). Recent advances in photocatalytic treatment of pollutants in aqueous media. Environ. Int..

[6] Xiao J., Xie Y., Cao H., Wang Y., Zhao Z. (2015). g-C_3_N_4_–triggered super synergy between photocatalysis and ozonation attributed to promoted OH generation. Catal. Commun..

[7] Weon S., Choi W. (2016). TiO_2_ nanotubes with open channels as deactivation-resistant photocatalyst for the degradation of volatile organic compounds. Environ. Sci. Technol..

[8] Fei J., Li J. (2015). Controlled preparation of porous TiO_2_-Ag nanostructures through supramolecular assembly for plasmon-enhanced photocatalysis. Adv. Mater..

[9] Pu C.C., Wan J., Liu E.Z., Yin Y.C., Li J., Ma Y.N., Fan J., Hu X.Y. (2017). Two-dimensional porous architecture of protonated GCN and reduced graphene oxide via electrostatic self-assembly strategy for high photocatalytic hydrogen evolution under visible light. Appl. Surf. Sci..

[10] Zhu B., Zhang J., Jiang C., Cheng B., Yu J. (2017). First principle investigation of halogen-doped monolayer g-C_3_N_4_ photocatalyst. Appl. Catal. B Environ..

[11] Wen J.Q., Xie J., Chen X.B., Li X. (2017). A review on g-C_3_N_4_-based photocatalysts. Appl. Surf. Sci..

[12] Zheng Y., Zhang Z., Li C., Proulx S. (2016). Surface hydroxylation of graphitic carbon nitride: Enhanced visible light photocatalytic activity. Mater. Res. Bull..

[13] Ong W.J., Tan L.L., Ng Y.H., Yong S.T., Chai S.P. (2016). Graphitic carbon nitride (g-C_3_N_4_)-based photocatalysts for artificial photosynthesis and environmental remediation: Are we a step closer to achieving sustainability?. Chem. Rev..

[14] Shi L., Chang K., Zhang H.B., Hai X., Yang L.Q., Wang T., Ye J.H. (2016). Drastic enhancement of photocatalytic activities over phosphoric acid protonated porous g-C_3_N_4_ nanosheets under visible light. Small.

[15] Yu X., Ng S.F., Putri L.K., Tan L.L., Mohamed A.R., Ong W.J. (2021). Point-defect engineering: Leveraging imperfections in graphitic carbon nitride (g-C_3_N_4_) photocatalysts toward artificial photosynthesis. Small.

[16] Hu J.S., Zhang P.F., An W.J., Liu L., Liang Y.H., Cui W.Q. (2019). In-situ Fe-doped g-C_3_N_4_ heterogeneous catalyst via photocatalysis-Fenton reaction with enriched photocatalytic performance for removal of complex wastewater. Appl. Catal. B Environ..

[17] Jiang L.B., Yuan X.Z., Pan Y., Liang J., Zeng G.M., Wu Z.B., Wang H. (2017). Doping of graphitic carbon nitride for photocatalysis: A review. Appl. Catal. B Environ..

[18] Guo S.E., Tang Y.Q., Xie Y., Tian C.G., Feng Q.M., Zhou W., Jiang B.J. (2017). P-doped tubular g-C_3_N_4_ with surface carbon defects: Universal synthesis and enhanced visible-light photocatalytic hydrogen production. Appl. Catal. B Environ..

[19] Bao Y., Chen K. (2018). Novel Z-scheme BiOBr/reduced graphene oxide/protonated g-C_3_N_4_ photocatalyst: Synthesis, characterization, visible light photocatalytic activity and mechanism. Appl. Surf. Sci..

[20] Tan Y., Shu Z., Zhou J., Li T., Wang W., Zhao Z. (2018). One-step synthesis of nanostructured g-C_3_N_4_/TiO_2_ composite for highly enhanced visible-light photocatalytic H_2_ evolution. Appl. Catal. B Environ..

[21] Zhu W., Gao X., Li Q., Li H., Chao Y., Li M., Mahurin S.M., Li H., Zhu H., Dai S. (2016). Controlled gas exfoliation of boron nitride into few-layered nanosheets. Angew. Chem. Int. Ed..

[22] Zhang J., Chen X., Takanabe K., Maeda K., Domen K., Epping J.D., Fu X., Antonietti M., Wang X. (2010). Synthesis of a carbon nitride structure for visible-light catalysis by copolymerization. Angew. Chem. Int. Ed..

[23] Novoselov K.S., Geim A.K., Morozov S.V., Jiang D., Zhang Y., Dubonos S.V., Grigorieva I.V., Firsov A.A. (2004). Electric field effect in atomically thin carbon films. Science.

[24] Luo J., Chen Q., Dong X. (2015). Prominently photocatalytic performance of restacked titanate nanosheets associated with H_2_O_2_ under visible light irradiation. Powder Technol..

[25] Chen Q., Luo J., Tao Y., Dong X. (2015). Free-standing films of titanate nanosheets as efficient visible-light-driven photocatalysts for environmental application. Mater. Lett..

[26] Dong X., Cheng F. (2015). Recent development in exfoliated two-dimensional g-C_3_N_4_ nanosheets for photocatalytic applications. J. Mater. Chem. A.

[27] Niu P., Zhang L., Liu G., Cheng H. (2012). Graphene-like carbon nitride nanosheets for improved photocatalytic activities. Adv. Funct. Mater..

[28] Yan J., Han X.X., Qian J.J., Liu J.Y., Dong X.P., Xi F.N. (2017). Preparation of 2D graphitic carbon nitride nanosheets by a green exfoliation approach and the enhanced photocatalytic performance. J. Mater. Sci..

[29] Miao H., Zhang G.W., Hu X.Y., Mu J.L., Han T.X., Fan J., Zhu C., Song L., Bai J., Hou X. (2017). A novel strategy to prepare 2D g-C_3_N_4_ nanosheets and their photoelectrochemical properties. J. Alloys Compd..

[30] Xu J., Zhang L., Shi R., Zhu Y. (2013). Chemical exfoliation of graphitic carbon nitride for efficient heterogeneous photocatalysis. J. Mater. Chem. A.

[31] Liu J., Zhang T., Wang Z., Dawson G., Chen W. (2011). Simple pyrolysis of urea into graphitic carbon nitride with recyclable adsorption and photocatalytic activity. J. Mater. Chem..

[32] Kang S.F., Zhang L., Yin C.C., Li Y.G., Cui L.F., Wang Y.G. (2017). Fast flash frozen synthesis of holey few-layer g-C_3_N_4_ with high enhancement of photocatalytic reactive oxygen species evolution under visible light irradiation. Appl. Catal. B Environ..

[33] Yang Y., Geng L., Guo Y., Meng J., Guo Y. (2017). Easy dispersion and excellent visible-light photocatalytic activity of the ultrathin urea-derived g-C_3_N_4_ nanosheets. Appl. Surf. Sci..

[34] Zhang Y., Zhou Z., Shen Y., Zhou Q., Wang J., Liu A., Liu S., Zhang Y. (2016). Reversible assembly of graphitic carbon nitride 3D network for highly selective dyes absorption and regeneration. ACS Nano.

[35] Wang F.L., Chen P., Feng Y.P., Xie Z.J., Liu Y., Su Y.H., Zhang Q.X., Wang Y.F., Yao K., Lv W.Y. (2017). Facile synthesis of N-doped carbon dots/g-C_3_N_4_ photocatalyst with enhanced visible-light photocatalytic activity for the degradation of indomethacin. Appl. Catal. B Environ..

[36] Wang Y., Wang H., Chen F., Cao F., Zhao X., Meng S., Cui Y. (2017). Facile synthesis of oxygen doped carbon nitride hollow microsphere for photocatalysis. Appl. Catal. B Environ..

[37] Xiao J., Xie Y., Nawaz F., Jin S., Duan F., Li M., Cao H. (2016). Super synergy between photocatalysis and ozonation using bulk g-C_3_N_4_ as catalyst: A potential sunlight/O_3_/g-C_3_N_4_ method for efficient water decontamination. Appl. Catal. B Environ..

[38] Fan C., Feng Q., Xu G., Lv J., Zhang Y., Liu J., Qin Y., Wu Y. (2018). Enhanced photocatalytic performances of ultrafine g-C_3_N_4_ nanosheets obtained by gaseous stripping with wet nitrogen. Appl. Surf. Sci..

[39] Li H.J., Sun B.W., Sui L., Qian D.J., Chen M. (2015). Preparation of water-dispersible porous g-C_3_N_4_ with improved photocatalytic activity by chemical oxidation. Phys. Chem. Chem. Phys..

[40] Yang X., Qian F., Zou G., Li M., Lu J., Li Y., Bao M. (2016). Facile fabrication of acidified g-C_3_N_4_/g-C_3_N_4_ hybrids with enhanced photocatalysis performance under visible light irradiation. Appl. Catal. B Environ..

[41] Cheng F., Wang H., Dong X. (2015). The amphoteric properties of g-C_3_N_4_ nanosheets and fabrication of their relevant heterostructure photocatalysts by an electrostatic re-assembly route. Chem. Commun..

[42] Behera A., Kandi D., Majhi S.M., Martha S., Parida K.M. (2018). Facile synthesis of ZnFe_2_O_4_ photocatalysts for decolourization of organic dyes under solar irradiation. Beilstein J. Nanotechnol..

[43] Meng S., Ye X., Ning X., Xie M., Fu X., Chen S. (2016). Selective oxidation of aromatic alcohols to aromatic aldehydes by BN/metal sulfide with enhanced photocatalytic activity. Appl. Catal. B: Environ..

